# Eugenol-hyperactivated nymphs of *Triatoma infestans* become intoxicated faster than non-hyperactivated nymphs when exposed to a permethrin-treated surface

**DOI:** 10.1186/s13071-018-3146-4

**Published:** 2018-11-03

**Authors:** Mercedes María Noel Reynoso, Alejandro Lucia, Eduardo Nicolás Zerba, Raúl Adolfo Alzogaray

**Affiliations:** UNIDEF-CITEDEF-CONICET- CIPEIN, Juan B. de La Salle 4397, (1603) Villa Martelli, Buenos Aires, Argentina

**Keywords:** *Triatoma infestans*, Triatominae, Eugenol, Permethrin, Hyperactivity, Increased intoxication, Insecticide pick up, Chagas disease

## Abstract

**Background:**

Eugenol is a botanical monoterpene that hyperactivates the blood-sucking bug *Triatoma infestans*, and permethrin is a pyrethroid with a strong triatomicide effect. In the present work, we tested the hypothesis that eugenol-hyperactivated nymphs of *T. infestans* pick up more insecticide, and then become intoxicated faster, than non-hyperactivated nymphs when exposed to a permethrin-treated surface.

**Results:**

Values of knockdown time 50% (KT50) for third-instar *T. infestans* exposed to a paper impregnated with permethrin were obtained under the following situations: (a.i.) immediately after topical application of eugenol (KT50: 66.75 min for acetone pre-treated controls, and 46.27 min for eugenol pre-treated nymphs); (a.ii.) 30 min after topical application of eugenol (KT50: 66.79 min for controls, and 66.79 min for eugenol pre-treated nymphs); (b) simultaneously with exposure to eugenol vapors (KT50: 51.90 min for controls, and 39.5 min for nymphs exposed to an eugenol-treated filter paper); and (c) immediately after an injection of eugenol (on average, controls were knocked down after 63.00 min, whereas nymphs injected with eugenol were knocked down after 65.30 min). In other experimental series, the distance traveled (DT) by nymphs exposed to eugenol was quantified in the same situations previously described, but without exposure to permethrin. In (a.i.), the DT in interval 0–30 min after topical application of eugenol was 487.00 (control) and 1127.50 (eugenol) cm; in (a.ii.), the DT in the interval 31–60 min after topical application was 336.75 (control) and 256.75 (eugenol) cm; in (b), DT was 939.08 (control) and 1048.53 (eugenol) cm; and in (c), it was 589.20 (control) and 700.00 (eugenol) cm. The KT50 values for permethrin decreased significantly in situations (a.i.) and (b), and eugenol only produced a significant hyperactivity in the same situations. Finally, the amount of permethrin picked up by non-hyperactivated and hyperactivated nymphs exposed to a film of permethrin was quantified by gas chromatography. Non-hyperactivated nymphs picked up 0.34 μg/insect of permethrin, while hyperactivated nymphs picked up 0.65 μg/insect.

**Conclusion:**

Results support the hypothesis that eugenol-hyperactivated nymphs of *T. infestans* pick up more insecticide, and then become intoxicated faster, than non-hyperactivated nymphs when exposed to a permethrin-treated surface.

## Background

Eugenol is a botanical monoterpene present in the essential oils of clove, cinnamon, nutmeg, basil and other plants [1]. Its very low toxicity in mammals allows it to be used in odontology (for surgical dressings, temporary fillings, pulp capping agents and cavity liners), as a food flavoring and in perfumes [2]. It also shows insecticidal and repellent activities in several insect species [3–7]. An almost unexplored sublethal effect of monoterpenes consists in the modification of insect locomotor activity [8, 9]. For example, eugenol hyperactivates nymphs of the blood-sucking bug *Triatoma infestans*, vector of *Trypanosoma cruzi*, the causal agent of Chagas disease in Latin America [8]. The primary sites of action of the vast majority of insecticidal monoterpenes are unknown, but there is evidence suggesting that eugenol is a ligand of the octopamine receptor of the insects’ nervous system [10].

When an organism is exposed to a mixture of two or more substances, their biological activity can be modified by the presence of the other compound. As a consequence of their interaction, the effect of the resulting combination can be higher or lower than the addition of the effects of each substance alone (synergism and antagonism, respectively) [11]. Both types of interactions have been observed in binary combinations of monoterpenes [12–15].

The classical concept of toxicological interactions considers the processes of absorption, distribution, metabolism, reaching sites of action and excretion of the components of the mixtures [16]. Some of these processes have been suggested as responsible for the toxicological interactions observed between monoterpenes [17–19]. In the cabbage looper, *Trichoplusia ni*, topical application of a mixture of *p*-cymene and thymol has a synergic effect since the first substance increases cuticle penetration, increasing the toxicity of the second substance [14, 20].

Other interactions involve changes in insect behavior. Incorporation of volatile host-plants increased the attraction of the boll weevil, *Anthonomus grandis*, and the palm weevil, *Rhynchophorus ferrugineus*, to their respective aggregation pheromones [21, 22]. In these cases, the behavioral response of insects exposed to a mixture of volatile substances was greater than the response to the components applied individually. This type of interaction has been named “behavioral synergism” [23].

The behavior of an insect can modify its exposure to a surface treated with insecticide. For example, hyperactive larvae of the tobacco budworm, *Heliothis virescens* picked up more permethrin from a treated surface than less active larvae of the same species [24].

Considering the information summarized above, the aim of this work was to test the hypothesis that eugenol-hyperactivated nymphs of *T. infestans* become intoxicated faster than non-hyperactivated nymphs when exposed to a permethrin-treated surface.

## Methods

### Biological material

We used third-instar nymphs of *T. infestans*, 1–14 days-old and starved after molt, from a colony at the Centro de Investigaciones de Plagas e Insecticidas (UNIDEF-CITEDEF-CONICET-CIPEIN, Buenos Aires, Argentina). This colony was established from a sample of insects donated by the Instituto Nacional de Parasitología “Dr. Mario Fatala Chaben” (Buenos Aires, Argentina). Insects were reared at a temperature of 25 ± 2 °C, 60–90% relative humidity and under a 12:12 h (L:D) photoperiod. Insects were fed on pigeon blood once per week according to a protocol approved by the Institutional Animal Care and Use Committee of CIPEIN (IACUC/CICUAL 1531/13).

### Chemicals

Permethrin (94.5%) was provided by Chemotecnica S.A. (C. Spegazzini, Buenos Aires, Argentina), eugenol (99%) was purchased from Sigma-Aldrich (Buenos Aires, Argentina), acetone (technical grade) from Merck (Darmstadt, Germany) and silicone oil (556 cosmetic grade fluid) from Daltosur S.R.L. (Buenos Aires, Argentina).

We used eugenol due to its reported effect on the locomotor activity of *T. infestans* nymphs [8]. On the other hand, we chose permethrin because, in addition to its proved lethal effect on *T. infestans* [25], we found that the concentrations needed for our toxicity assays did not modify the locomotor activity of the nymphs. This was a key requirement, as in order to test our hypothesis we needed only eugenol to produce hyperactivity.

### Bioassays of toxicity

#### Topical application of eugenol followed by exposure to a permethrin-treated surface

A disc of filter paper 11 cm in diameter (102 FAST, Hangzhou Xinxing Paper Industry & Co. Ltd., Funyang, China) was impregnated with 0.5 ml permethrin dissolved in an acetone:silicone (1:1) solution (1840 mg/m^2^). The solvent was left to evaporate for an hour and then a glass ring (4 cm high and 10 cm in diameter) was placed over the paper disc.

Using a microsyringe (Hamilton, Reno, NE), 1 μl of eugenol dissolved in acetone was dropped onto the dorsal side of the abdomen of ten nymphs (0.1 μg/insect). They were then placed on the disc of filter paper impregnated with permethrin (a.i) immediately or (a.ii) 30 min after the topical application of eugenol. As controls, ten nymphs were treated with acetone alone in each case. The number of knockdown individuals was registered every 5 min. A nymph was considered knocked down if it remained in the same place for 30 s after being gently touched with a soft pair of tweezers (controls walked away immediately after being touched). Three independent replicates were carried out and each included a group of ten nymphs exposed to a filter paper impregnated only with the solvent. Knockdown time 50% (KT50) values were calculated with the results.

#### Simultaneous exposure to eugenol vapors and a permethrin-treated surface

**Fig. 1:**
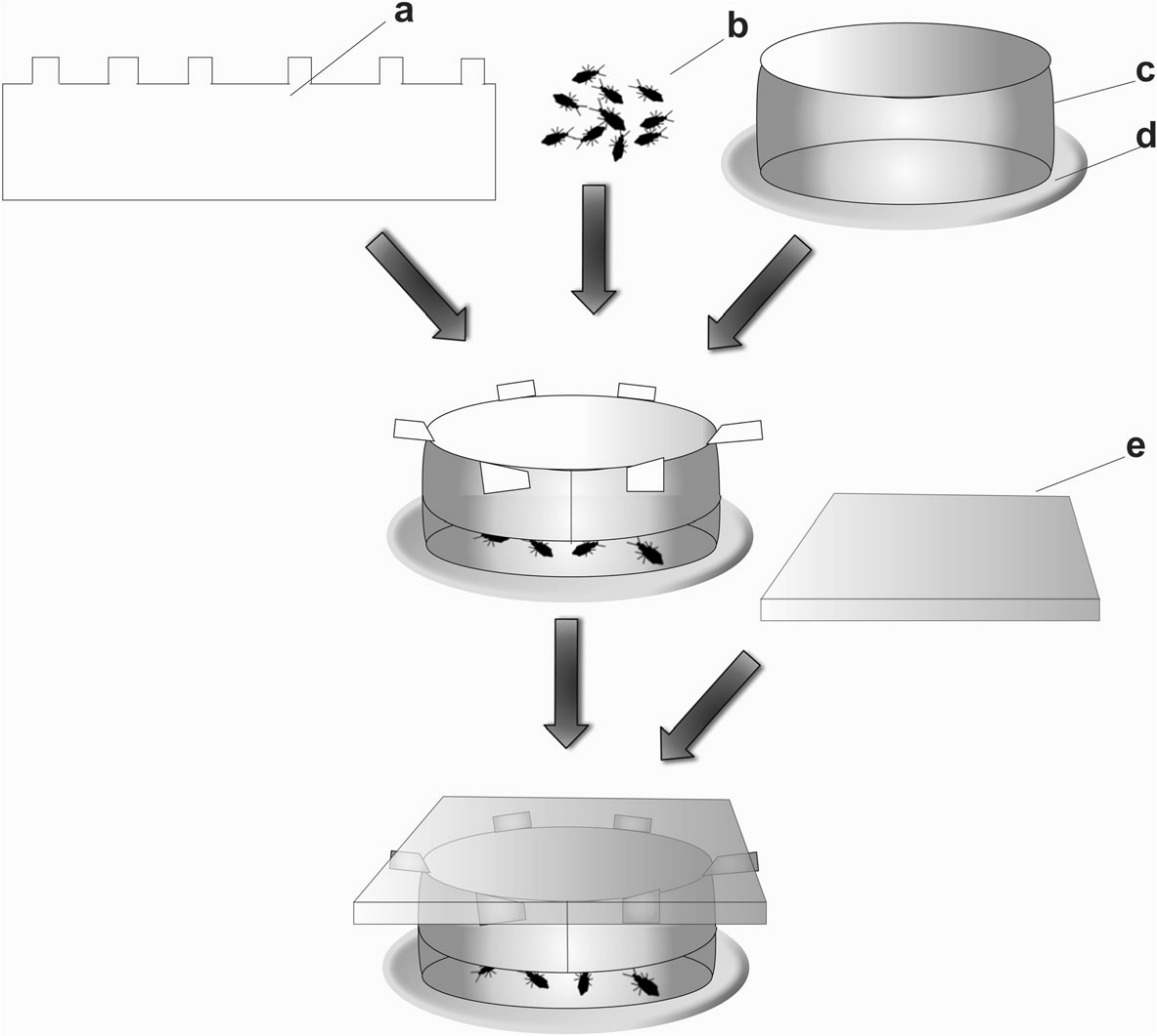
Experimental arena used for simultaneous exposure of *T. infestans* nymphs to eugenol (vapors) and permethrin (contact): eugenol-impregnated piece of rectangular filter paper with six square lapels (**a**), *T. infestans* nymphs (**b**), glass ring (**c**), filter paper impregnated with permethrin (**d**), and glass square (**e**)

The source of vapors was a piece of rectangular filter paper impregnated with eugenol (3.0 × 29.1 cm) (Fig. 1). One of the longer sides of the rectangle had six square lapels (0.5 × 0.5 cm) separated by 4.3 cm, that were turned up at a 90° angle. The surface of the rectangle was treated with 0.5 ml of eugenol dissolved in acetone (33 μg/cm^2^). Once the solvent had evaporated, the inner side of the glass ring was lined with the paper rectangle, leaving the lapels to hang over the upper side of the ring. Thus, when the glass ring was placed over the circle of filter paper impregnated with permethrin, the bottom side of the paper rectangle hung 1 cm above the filter paper and was out of reach from the ten nymphs that were subsequently placed inside the ring. Finally, the upper side of the ring was covered with a glass square (10 × 10 cm) to create a micro-atmosphere with eugenol. Four independent replicates were carried out and each included a group of ten nymphs exposed to a piece of rectangular filter paper treated with acetone alone.

#### Injection of eugenol followed by exposure to a permethrin-treated surface

The upper side of a slide was covered by double-sided adhesive tape and a nymph was placed in a supine position and attached dorsally. Using a microsyringe and a binocular Nikon SMZ-10 10× magnifying glass (Tokyo, Japan), 0.2 μl of eugenol dissolved in acetone was injected into the nymph (2 ng/insect). The injection was performed in the left pleura of the first abdominal segment using a microsyringe (Hamilton, Reno, NE, USA). The nymph was then gently unstuck from the adhesive tape and placed in the center of a permethrin-impregnated filter paper disc. A nymph injected with acetone alone was used as control. As the immobilization and injection of the nymphs took longer than a topical application, in this series of experiments nymphs were individually exposed to a permethrin-treated filter paper. The time until each nymph was knocked down was registered. Each treatment was independently repeated ten times.

### Bioassays of locomotor activity

Experiments were carried out inside a wooden box coated with melamine (1.0 × 0.5 × 0.5 m). The experimental arena was a filter paper disc (11 cm in diameter) (102 FAST; Hangzhou Xinxing Paper Industry & Co. Ltd.) placed on the floor of the box. A glass ring (4 cm high and 10 cm in diameter) was placed over the paper disc. A video camera was located 20 cm above the experimental arena (HD Webcam C525, Logitech, Lausanne, Switzerland) and connected to a personal computer. The camera had a resolution of 640 × 480 pixels with an acquisition and image processing speed of 30 frames/second. Lighting was provided by a cold light bulb (22 watts; Luxa, Shanghai, China) placed in the center of the top of the box.

A nymph was placed on the center of the filter paper and its movement was registered for 60 min. This experimental time allowed determining the duration of the effect of eugenol on locomotor activity and observing the way nymphs behaved once the hyperactive effect disappeared.

The video was analyzed using EthoVision XT 10.0 software [26]. This program quantifies the distance an insect moves expressed in cm.

The four experimental series described below (a’-d’) were carried out using the same doses and concentrations of eugenol and permethrin as used in the toxicity bioassays. After testing that there were no variations in nymph locomotor activity between 11 am and 15 pm, trials were always performed within that time frame.

- (a’) Topical application of eugenol. Three independent replicates were performed, each including a nymph treated with acetone alone and another one treated with 0.1 μg eugenol/insect;
- (b’) Exposure to eugenol vapors. Six independent replicates were carried out, each including a nymph exposed to a piece of rectangular filter paper treated with acetone alone and another one exposed to a piece of rectangular filter paper impregnated with a solution of eugenol in acetone (33 μg/cm^2^);
- (c’) Eugenol injection. One nymph was injected with acetone alone and another with 2 ng eugenol/insect. Each treatment was independently repeated ten times;
- (d’) Exposure to a paper filter disc impregnated with permethrin. Three independent replicates were carried out, each including a nymph exposed to a disc of filter paper treated with solvent alone and another one exposed to a disc of filter paper treated with permethrin (1840 mg/m^2^).

Each of these four experimental series included one or two untreated control groups as follows: in series (a’), nymphs with no topical application; in (b’), nymphs exposed to an untreated piece of rectangular filter paper; in (c’), a group of nymphs pricked with the microsyringe needle but not injected with any substance, and a group of non-pricked nymphs; and in (d’), nymphs exposed to an untreated filter paper disc.

### Extraction of the permethrin picked up by the nymphs

The procedure published by Pennell et al. [27] was adopted in this study with major modifications. Five hundred microliters of permethrin in acetone (0.035 g/ml) were applied on a circle of filter paper to obtain a concentration of 1840 mg/m^2^. After solvent evaporation, the filter paper was placed on a horizontal surface and a glass ring was placed over it. Then, a group of ten nymphs topically pre-treated with 0.1 μg/insect of eugenol in acetone was placed on the permethrin-treated filter paper. After 10 min, the insects were transferred to a glass vial (2 ml) and fully immersed in 0.5 ml of acetone. The glass vial was gently shaken for 2 min after which the insects were discarded. Finally, an additional volume of acetone was added to the vial to complete 1 ml.

### GC-MS analysis for determining the quantity of permethrin picked up

The analysis of permethrin was performed using a GC-2010 instrument coupled with a QP2010 mass spectrometer detector (Shimadzu, Kyoto, Japan) in the electron impact mode (70 eV). Samples were analyzed following the methodology used by Pinheiro et al. [28]. An apolar GC capillary column DB-5 (30 m × 0.25 mm i.d. and 0.25 μm coating thickness; J&W Scientific, Folsom, CA, USA) was used. The GC column temperature was maintained at 60 °C for 1 min, then programmed to increase from 60 to 150 °C at a rate of 20 °C/min, held for 4 min and then increased to 290 °C at a rate of 15 °C/min. This final temperature was held for 5.17 min. The injector was held at 280 °C. Helium (99.99%) was used as the carrier gas at a 1.2 ml/min caudal. The detector temperature was 250 °C and all injections were performed in the splitless mode (sampling time: 2 min). The volume of sample injected was 1 μl. The mass spectrometer scanned mass range m/z 40 and 400 was used for quantitative determinations of permethrin. For quantitative determination using selective ion monitoring (SIM), permethrin was identified by an ion of mass 183 (retention time: 20.15 min). The quantification was performed by calculating the absolute peak areas and a calibration curve was constructed by plotting the peak area of technical permethrin (purity 95.4%; cis: 52.4) *versus* permethrin concentration using a linear regression model (R^2^: 0.99). With this calibration curve, the amount of permethrin in 1 ml (volume used for the extraction of 10 individuals) was obtained. These values were divided by 10 to obtain the amount of permethrin recovered per nymph (permethrin recovered expressed as μg per insect) of the insects previously treated by topical application of eugenol (treatment) or acetone alone (control).

In summary, the experiment for determining the amount of permethrin picked up by the nymphs consisted of two parts: (a) extraction of the permethrin picked up by the nymphs, and (b) GC-MS analysis for determining the permethrin picked up. This experiment (a + b) was performed five times. Thus, the values of the amount of permethrin picked up are representative of five independent experiments. Additionally, as the error was greater than in a routine analysis (by total scan mode), each sample was injected three times and the resulting values were used to calculate the total amount of permethrin. The average variation coefficient obtained for 30 injections by GC-MS was 18.76%.

### Statistical analyses

Values of knockdown time 50% (KT50) and their corresponding 95% confidence intervals (95% CI) were calculated with the software for correlated data developed by Throne et al. [29]. Differences among KT50 values were consider significant if their 95% CI did not overlap. The results of the locomotor activity bioassays were analyzed using ANOVA, and those from the permethrin pick up quantification using Student’s t-test.

## Results

The toxicity of permethrin applied as a film on filter paper (1840 mg/m^2^) was quantified in nymphs previously treated topically with eugenol (0.1 μg/insect) (Table 1). When nymphs were exposed to permethrin immediately after the pre-treatment with eugenol, their KT50 (46.27 min) was significantly lower compared to the control group (66.75 min) (*P* < 0.05). However, when the nymphs were exposed to permethrin 30 min after the pre-treatment, no significant differences were observed between the KT50 values of the group treated with eugenol (66.79 min) and the control group (66.79 min).

**Table 1:** Knockdown time 50% (KT50) for permethrin in *T. infestans* nymphs topically pre-treated with eugenol

Pre-treatment (topical application)	Time before exposure to a permethrin-treated filter paper (min)	KT50 for permethrin (min)	95% CI	Slope ± SE
Acetone	0	66.75^a^	60.43–74.43	8.99 ± 1.51
Eugenol (0.1 μg/insect)	0	46.27^b^	40.90–52.29	7.40 ± 1.15
Acetone	30	66.79^a^	62.16–71.94	12.68 ± 2.07
Eugenol (0.1 μg/insect)	30	66.79^a^	61.87–72.32	11.90 ± 1.98

*Abbreviations*: *KT50*, knockdown time 50%; *95% CI*, 95% confidence interval; *SE*, standard error

*Notes*: Permethrin was applied as a film on filter paper (1840 mg/m^2^). KT50 values followed by different letters are significantly different (based on their respective 95% CI not overlapping; *P* < 0.05)

**Fig. 2:**
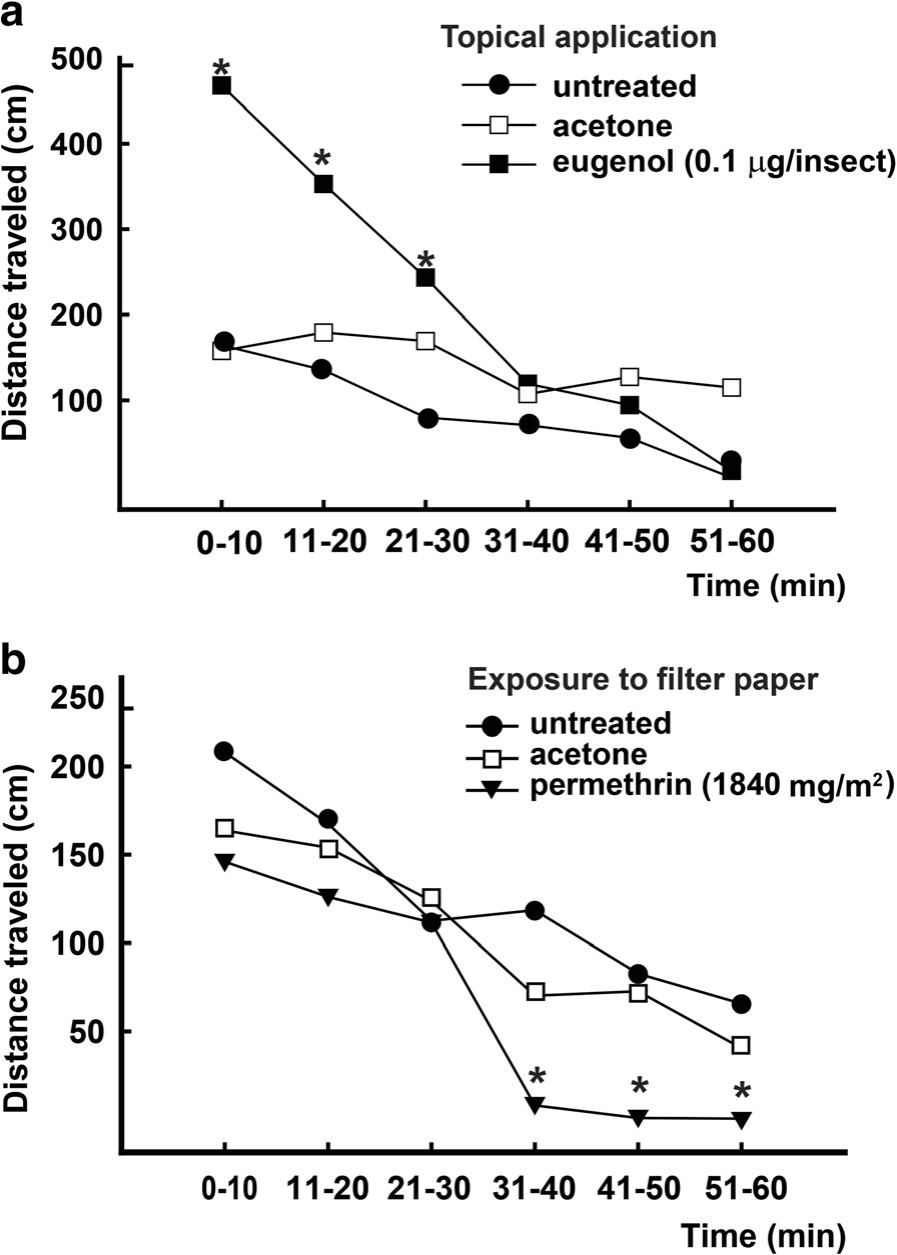
Locomotor activity of *T. infestans* nymphs topically treated with eugenol or exposed to permethrin. **a** Nymphs were topically treated with acetone alone (control, empty squares) or a solution of eugenol in acetone (filled squares). **b** Nymphs were exposed to a filter paper impregnated with acetone alone (empty squares) or a solution of permethrin in acetone (filled triangles). Each symbol represents the mean of four independent replicates. Means in each time interval were analyzed using ANOVA. Symbols marked with an asterisk are significantly different from both untreated and acetone controls (*P* < 0.05)

The second experiment assessed the effect of eugenol and permethrin on nymphs locomotor activity (Fig. 2). The same doses used in the previous experiment were evaluated. Topical application of eugenol significantly hyperactivated the nymphs but only for 30 min (for time interval 0–10 min, *F*_(2, 6)_ = 169.8, *P* < 0.001; 11–20 min, *F*_(__2, 6__)_ = 15.7, *P* = 0.001; 21–30 min, *F*_(__2,__6__)_ = 6.22, *P* = 0.02) (Fig. 2a). Exposure to filter paper impregnated with permethrin did not initially affect the locomotor activity of nymphs, but during the last minutes of the assay the activity decreased significantly (for the time interval 0–10 min: *F*_(2, 6)_ = 4.2, *P* = 0.052; 11–20 min: *F*_(2, 6)_ = 1.4, *P* = 0.29; 21–30 min: *F*_(2, 6)_ = 0.37, *P* = 0.70; 31–40 min: *F*_(2, 6)_ = 9.95, *P* = 0.005; 41–50 min: *F*_(2, 6)_ = 27.3, *P* < 0.001; 51–60 min: *F*_(2, 6)_ = 8.83, *P* = 0.008) (Fig. 2b). This decrease in locomotor activity coincided with the appearance of spasms, convulsions, and paralysis of the third pair of legs. These are the first symptoms of intoxication with pyrethroids, which manifest before knockdown.

**Fig. 3:**
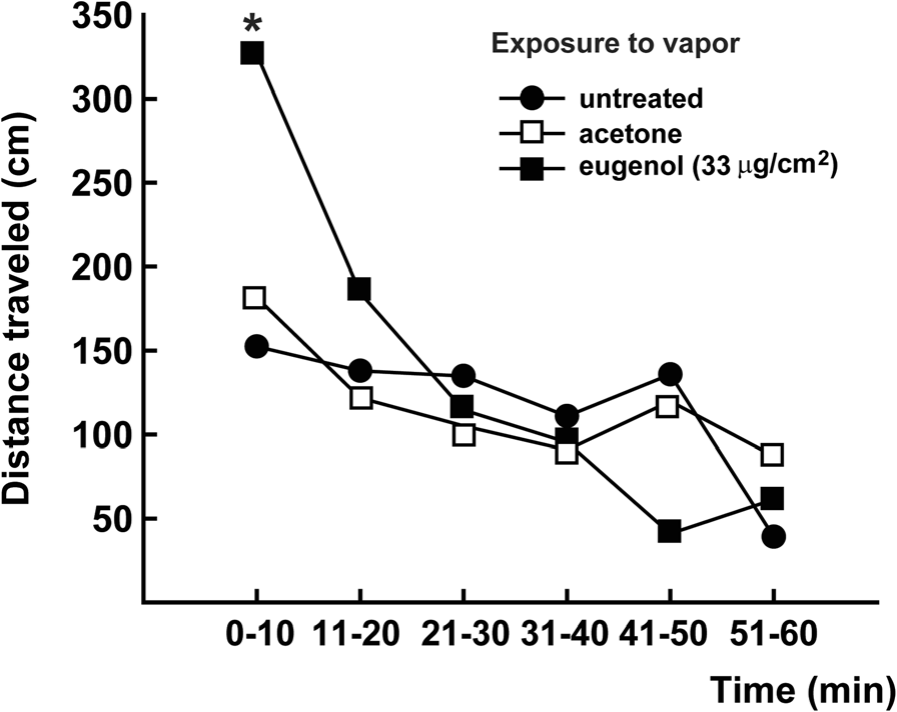
Locomotor activity of *T. infestans* nymphs exposed to vapors of eugenol. Nymphs were exposed to a filter paper impregnated with acetone (control, empty squares) or a solution of eugenol in acetone (filled squares). Each symbol represents the mean of four independent replicates. Means in each time interval were analyzed using ANOVA. Symbols marked with an asterisk are significantly different from both untreated and acetone controls (*P* < 0.05)

Next, we assessed the toxicity of permethrin (1840 mg/m^2^) on nymphs simultaneously exposed to eugenol vapors released by an impregnated paper (33 μg/cm^2^) (Table 2). The KT50 values of nymphs exposed to both substances (39.50 min) was significantly lower than the values for the control group (51.90 min) (*P* < 0.05). Additionally, exposure to eugenol vapors in the absence of permethrin only produced a significant increase in locomotor activity during the first 10 min (*F*_(2, 9)_ = 10.86, *P* = 0.001) (Fig. 3).

**Table 2:** Knockdown time 50% (KT50) for permethrin in *T. infestans* nymphs exposed simultaneously to eugenol vapor

Simultaneous exposure to:	KT50 (min)	95% CI	Slope ± SE
Acetone	51.90^a^	46.9–56.7	8.70 ± 1.39
Eugenol (33 μg/cm^2^)	39.50^b^	34.9–44.8	6.30 ± 0.89

*Abbreviations*: *KT50*, knockdown time 50%; *95% CI*, 95% confidence interval

*Notes*: Permethrin was applied as a film on filter paper (1840 mg/m^2^). KT50 values followed by different letters are significantly different (based on their respective 95% CI not overlapping; *P* < 0.05)

**Fig. 4:**
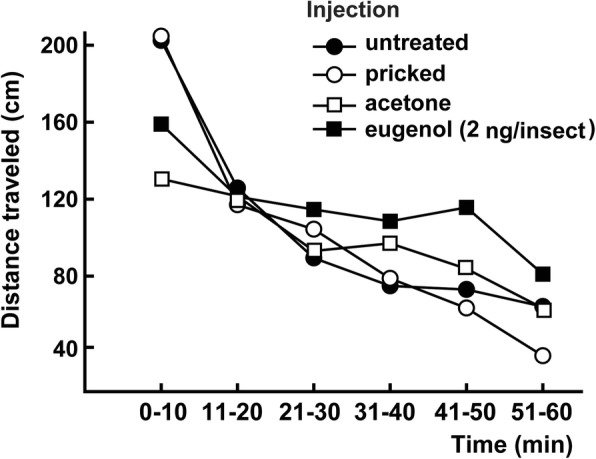
Locomotor activity of *T. infestans* nymphs injected with eugenol. Nymphs were injected with acetone alone (control, empty squares) or a solution of eugenol in acetone (filled squares). Each symbol represents the mean of ten independent repetitions. Means in each time interval were analyzed using ANOVA. No significant differences were detected in any case (*P* > 0.05)

We then determined the mean KT50 values of nymphs injected with eugenol (2 ng/insect) and immediately exposed to a paper disc impregnated with permethrin. On average, the KT50 value of nymphs injected with eugenol was 65.30 ± 4.80 min while the KT50 of nymphs injected with acetone alone was 63.0 ± 2.63 min. The difference between these values was not significant (*P* > 0.05), indicating that eugenol injection did not significantly modify the locomotor activity of nymphs compared to the control group (for the time interval 0–10 min: *F*_(3, 36)_ = 1.94, *P* = 0.14; 11–20 min: *F*_(3, 36)_ = 0.04, *P* = 0.99; 21–30 min: *F*_(3, 36)_ = 0.45, *P* = 0.72; 31–40 min: *F*_(3, 36)_ = 0.43, *P* = 0.73; 41–50 min: *F*_(3, 36)_ = 1.32, *P* = 0.28; 51–60 min: *F*_(3, 36)_ = 0.90, *P* = 0.45) (Fig. 4).

**Fig. 5:**
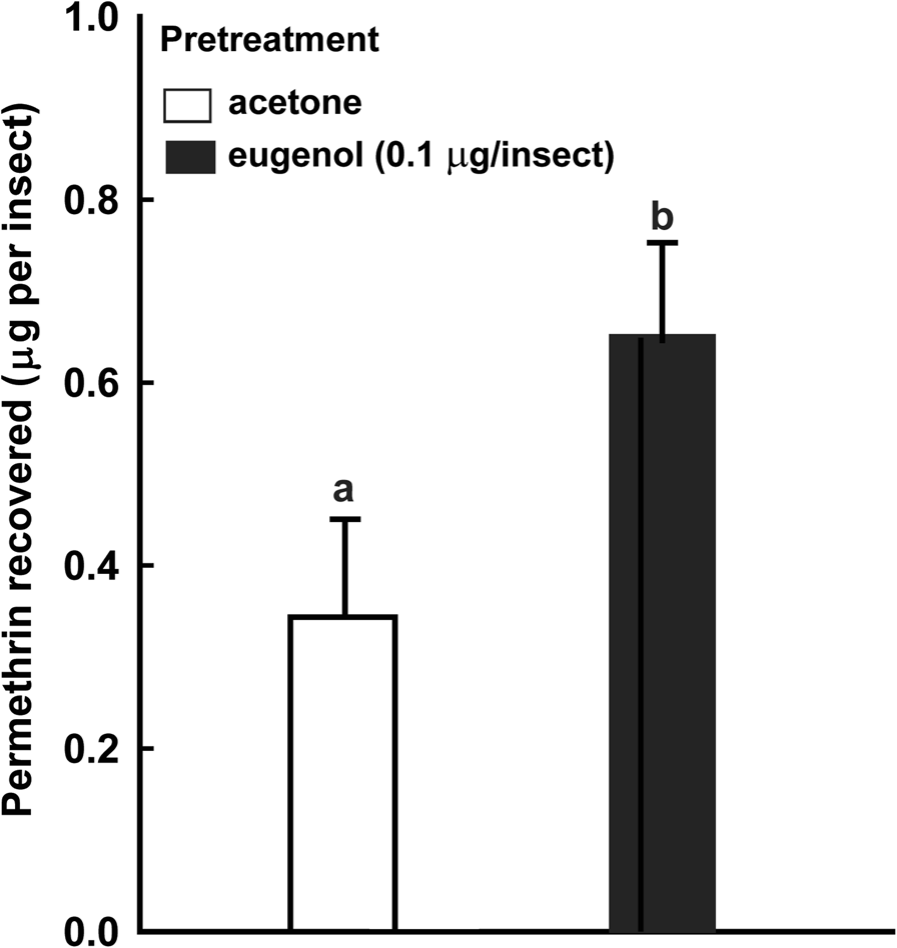
Permethrin picked-up by hyperactivated *T. infestans* from an impregnated filter paper. Nymphs were topically pre-treated with acetone alone (empty bar) or a solution of eugenol in acetone (filled bar), then exposed to a filter paper treated with permethrin (1840 mg/m^2^). Each bar represents the mean of five independent replicates. Vertical lines represent the SE. Bars marked with different letters are significantly different (Student’s t-test, *P* < 0.05)

Finally, we assessed the amount of permethrin picked up by nymphs treated topically with eugenol and immediately exposed to permethrin impregnated filter papers for 10 min (Fig. 5). Nymphs pre-treated with eugenol picked up significantly higher quantities of permethrin than individuals pre-treated with acetone alone (*t*_(8)_ = 8.17, *P* = 0.001).

## Discussion

In the present study, we report that nymphs of *T. infestans* hyperactivated by eugenol pick up more insecticide from a permethrin-treated surface, thus becoming intoxicated faster than non-hyperactivated nymphs.

We quantified knockdown values produced by permethrin in four different situations: (a.i) immediately after a topical application of eugenol; (a.ii) 30 min after a topical application of eugenol; (b) simultaneously with the exposure to eugenol vapors; and (c) immediately after eugenol injection. The KT50 values of permethrin were significantly lower than the controls in situations (a.i) and (b). Moreover, when eugenol was applied in the absence of permethrin, it only produced hyperactivity in those same two situations.

Locomotor activity is a complex characteristic that is directly or indirectly involved in almost all the activities of an insect and is regulated by a neurophysiological mechanism [30]. It has a very important role in survival and depends on both internal (age, hormonal state) and external (presence or absence of predators and conspecifics) factors [31–34]. Of particular interest is the recent discovery that infection with *T. cruzi* increases the locomotor activity of *R. prolixus*, whereas infection with *T. rangeli* causes a decrease [35].

Exposure to DDT and other insecticides increases the locomotor activity of insects [36–38]. Nymphs of *T. infestans* resistant to deltamethrin showed a reduced sensitivity to the hyperactivity produced by this insecticide when compared to deltamethrin-susceptible individuals [39]. Hyperactivity was also observed in insects exposed to components of essential oils [10], but it was only quite recently that this effect was quantified exposing *T. infestans* and *R. prolixus* to alcoholic monoterpenes [8, 9].

In the present study, the effect of eugenol on the locomotor activity of the nymphs varied considerably according to the form of administration. Topical application produced the highest effect, followed by exposure to vapors, whereas eugenol injection caused no modification in nymph locomotor activity.

The amount of compound that reaches an insect depends strongly on the type of exposure [40]. For example, insects exposed to an insecticidal film mainly pick up the compound by their legs, whereas topical application generates a more extensive distribution on the body cuticle [41].

In the present study, nymphs treated topically received a larger amount of eugenol than those exposed to vapors. Therefore, the difference observed in the effect on locomotor activity could be due to the fact that a higher dose produces a greater effect.

Why did eugenol fail to produce hyperactivity when injected? The dose applied (2 ng of eugenol/insect) might have been too low to modify the nymphs’ locomotor activity. In fact, hyperactivity by eugenol and other monoterpenes is dose-dependent in *T. infestans* and *R. prolixus* [8, 9]. We were unable to test higher doses because they rapidly produced symptoms of intoxication (lateral walking, proboscis extension, leg tremors). Applying a substance that hyperactivates the nymphs at a dose that is much lower than the threshold producing intoxication symptoms could help solve this issue.

On the other hand, the effect of eugenol on hyperactivity could be induced *via* the neurons of sensory structures on the cuticle that react to different environmental stimuli. If this is true, stimulation would not occur when the monoterpene is injected into the hemolymph. The very rapid manifestation of hyperactivity when nymphs were exposed to eugenol vapors (Fig. 4) provides evidence that supports this explanation.

The mode of action of most monoterpenes remains unknown. Eugenol is one of the few exceptions, as evidence suggests that its primary site of action is the octopaminergic receptor [10]. The octopaminergic system is widely distributed throughout the central and peripheral nervous systems of invertebrates, where it participates in many physiological and behavioral aspects [42]. Within the context of the present study, it is important to note that the octopaminergic system is involved in the initiation and maintenance of rhythmic behaviors such as walking [43–45]. Based upon this knowledge, an interaction between eugenol and octopaminergic receptors could explain the hyperactivity observed in our experiments.

After demonstrating that hyperactivated nymphs become intoxicated more rapidly than the non-hyperactivated ones when exposed to a permethrin-treated surface, we quantified the amount of insecticide permethrin picked up in both situations. For this we adapted two protocols, one used for extracting dieldrin picked up by mosquitoes exposed to a surface treated with this insecticide [27], and another used to quantify permethrin and other insecticides in water samples [28]. This way, we managed to detect minute quantities of permethrin picked up by the nymphs exposed to treated filter papers. Our results showed that the nymphs hyperactivated by eugenol picked up more permethrin than the non-hyperactivated ones.

As mentioned in Background, a similar result was observed by other authors working with two strains of *H. virescens*, one susceptible and the other resistant to permethrin [24]. When exposed to a permethrin-treated surface, larvae from the resistant strain were less active and picked up less insecticide, than larvae from the susceptible strain. This behavioral response was considered partially responsible for the resistance to permethrin.

In summary, here we have gathered a significant amount of evidence that support our hypothesis: eugenol-hyperactivated nymphs of *T. infestans* pick up more insecticide, and then become intoxicated faster, than non-hyperactivated nymphs when exposed to a permethrin-treated surface.

Future studies should investigate the effects of other monoterpenes as possible enhancers of the toxicity of different insecticides on triatomines. Mixtures of monoterpenes should also be evaluated, to establish whether synergistic interactions occur. The application of mixtures of monoterpenes and conventional insecticides under field conditions is a possibility that is worth exploring, since hyperactivity could increase the exposure to recently applied insecticides, increasing the efficacy of control. Another issue to be investigated is the effect of eugenol on the locomotor activity of both trypanosomes-infected and resistant to pyrethroids *T. infestans*. These studies could elucidate for the physiological basis of the hyperactivity induced by insecticides in insects.

## Conclusion

Results from toxicological, behavioral and analytical assays performed in this work support the hypothesis that eugenol-hyperactivated nymphs of *T. infestans* pick up more insecticide, and then become intoxicated faster, than non-hyperactivated nymphs when exposed to a permethrin-treated surface.

## Abbreviations

95% CI: 95% confidence interval
DT: Distance traveled
GC-MS: Gas chomatography - mass spectrometry
KT50: Knockdown time 50%
